# Inclusion Detection in Injection-Molded Parts with the Use of Edge Masking

**DOI:** 10.3390/s24227150

**Published:** 2024-11-07

**Authors:** Pawel Rotter, Maciej Klemiato, Dawid Knapik, Maciej Rosół, Grzegorz Putynkowski

**Affiliations:** 1 AGH University of Krakow, al. Mickiewicza 30, 30-059 Krakow, Poland; mkl@agh.edu.pl (M.K.); knapik@agh.edu.pl (D.K.); mr@agh.edu.pl (M.R.); 2 CBRTP S.A.—Centrum Badań i Rozwoju Technologii dla Przemysłu S.A., ul. Ludwika Waryńskiego 3A, 00-645 Warszawa, Poland; grzegorz.putynkowski@cbrtp.pl

**Keywords:** optical inspection, defect detection, image matching, object classification

## Abstract

The algorithm and prototype presented in the article are part of a quality control system for plastic objects coming from injection-molding machines. Some objects contain a flaw called inclusion, which is usually observed as a local discoloration and disqualifies the object. The objects have complex, irregular geometry with many edges. This makes inclusion detection difficult, because local changes in the image at inclusions are much less significant than grayscale changes at the edges. In order to exclude edges from calculations, the presented method first classifies the object and then matches it with the corresponding mask of edges, which is prepared off-line and stored in the database. Inclusions are detected based on the analysis of local variations in the surface grayscale in the unmasked part of the image under inspection. Experiments were performed on real objects rejected from production by human quality controllers. The proposed approach allows tuning the algorithm to achieve very high sensitivity without false detections at edges. Based on input from the controllers, the algorithm was tuned to detect all the inclusions. At 100% recall, 87% precision was achieved, which is acceptable for industrial applications.

## 1. Introduction

**Figure 1:**
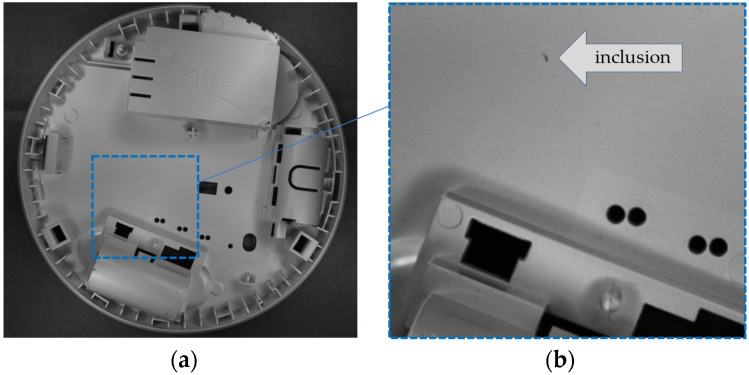
Example of object from the database (**a**) and its fragment with a visible inclusion (**b**).

This paper presents a method for detecting inclusions in plastic objects. Inclusions are small discolorations resulting from the manufacturing process that make the part defective and appear as blobs of a different color compared to the rest of the surface. An example of an inclusion in an injection-molded part is presented in Figure 1. The goal of the system is to replace human quality controllers who check each sample for inclusions. An important part of the algorithm is the exclusion of edges from calculations because it is based on the analysis of local grayscale variations in the image, and edges would cause false alarms. However, applying edge detectors directly to the image could classify some inclusions as edges, preventing their correct recognition. Therefore, a predefined edge mask aligned with the reference image was used. Matching the reference image with the camera image allows finding the transformation parameters to superimpose the edge mask onto the image.

### 1.1. Background and Related Work

There are many methods for defect detection in *homogeneous or textured surfaces*. A brief review of surface detection methods was conducted by a research group [1]. In particular, metal planar materials have garnered significant interest. An overview of defect detection methods specifically designed for these materials is presented in [2]. Classical methods are mostly based on texture analysis [3] and local intensity analysis. In this approach, methods such as regularity measures of the inspected surface [4], grayscale fluctuations [5], color variation [6], or fractal features [7] are used. Apart from the inspection of moving objects, there are also methods for the optical inspection of the conveyor belt itself. In this case, vision systems are used to detect longitudinal rips, belt deviation/deflections, and the overall offset using linear light sources and a line-array CCD camera [8] or a single camera and a line laser generator [9]. Many methods of defect detection are based on *deep neural networks*. A review of the latest developments in industrial defect detection based on machine vision and deep learning, including defect classification, localization, and segmentation is presented in [10]. The survey paper [11] presents different ways of classifying various efforts in the literature for surface defect detection using deep learning techniques: defect detection context, learning techniques, defect localization, and classification. An example of a quality control system for the assessment of products made by injection molding using the MLP neural network model is presented in [12]. In [13], a method based on the Faster Region-based Convolutional Neural Network for structural visual inspection is proposed to provide quasi-real-time simultaneous detection of multiple types of damage. In another approach, presented in [14], the detection of anomalies in images is based on the features extraction capabilities of convolutional neural networks. An interesting application to evaluate defects on the surface of the materials at the 3D level is presented in paper [15]. A DenseNet convolutional neural network is employed to identify defect types using a 3D point cloud of a designated defect area. These applications usually operate on images of surfaces, without using any knowledge about the object geometry and its natural irregularities. However, the robustness of such methods and defect types that can be detected are limited [10]. Moreover, training deep neural networks requires a very large number of labeled samples. The existing applications of deep networks for defect detection focus on specific types of industrial products where public databases of surface defects exist, for example, steel belts, rails, strip steel, silicon steel, aluminum profile, solar panels, roads, or fabric. For details, see the review article [16]. Such a database for the specific problem of injection-molded parts does not exist, and manufacturers can only provide a limited set of defective elements containing inclusions, which is insufficient to train a deep network. 

A common problem when analyzing surfaces is related to light reflections, which can be a source of false defect detection. For this reason, it is important to ensure a highly uniform source of light. Some methods for indoor lighting optimization exist, for example, an efficient numerical algorithm for computing light scattering by a coated sphere [17] or a method based on an improved artificial bee colony algorithm [18]. The uniformity of light can be verified using LED and indoor space lighting models [18], goniophotometers, or the fisheye camera method [19,20]. In the prototype described in Section 2, a special lighting dome was used in order to avoid light reflections and ensure uniformity of light; see Section 2.1 for the details. 

### 1.2. The Main Contribution

The complexity of the problem presented in the paper is related to the intricate geometry of the examined elements. As shown in Figure 1, the magnitude and size of the inclusion are very small compared to the changes in the image grayscale at the edges. Algorithms that could detect inclusions as local changes in the image intensity would mostly return false detections at intensity changes resulting from the geometry of the object. This is why, in the first step, the edges of the object should be localized in the image and excluded from calculations. A straightforward approach would use edge detection algorithms, such as the Canny edge detector [21] or the two-dimensional Gabor filter [22]. However, such filtering-based methods could not discriminate between weak edges and some flaws of the element, which would generate local, closed edges. Any flaws incorrectly classified as edges, and therefore excluded from further calculations, could not be detected. 

The proposed method takes advantage of knowledge about the objects stored in the database with 3D models and the resulting edge masks, prepared off-line based on the model. Identification of the object type and matching the corresponding 3D model with the camera image allows the calculation of the edge location. The edge calculation does not rely on any edge detector, so it does not depend on the distinctiveness of the edges in the camera image, nor the quality of the camera image. Edges are calculated faultlessly as the result of a geometrical transformation of the model.

The parts are produced based on CAD models, which are available at the stage of quality control. For some objects, there are differences between models and produced elements, which contain injection marks resulting from the technological process. For that reason, post-production 3D models were utilized. These models are obtained by scanning correctly produced samples using the GOM Atos Scanbox. In principle, 3D models are used at the post-production stage to control the correctness of product geometry. However, such models can also be helpful for the localization of the edges of the parts in order to exclude them from inclusion detection. 

The proposed method presents an advancement in the field of quality control for objects with complex geometries. By applying an off-line calculated edge mask, we can effectively exclude edges from the inclusion detection process, reducing false positives and increasing detection accuracy. This method does not require extensive datasets for training deep neural networks and can be implemented using existing CAD models and 3D scanning technologies. The prototype system was awarded a gold medal at Automaticon International Automation and Measurements Fair 2023 in Warsaw (https://www.automaticon.pl/zloty-medal-2023 (accessed on 4 November 2024)). 

## 2. Materials and Methods

### 2.1. The Laboratory Stand 

**Figure 2:**
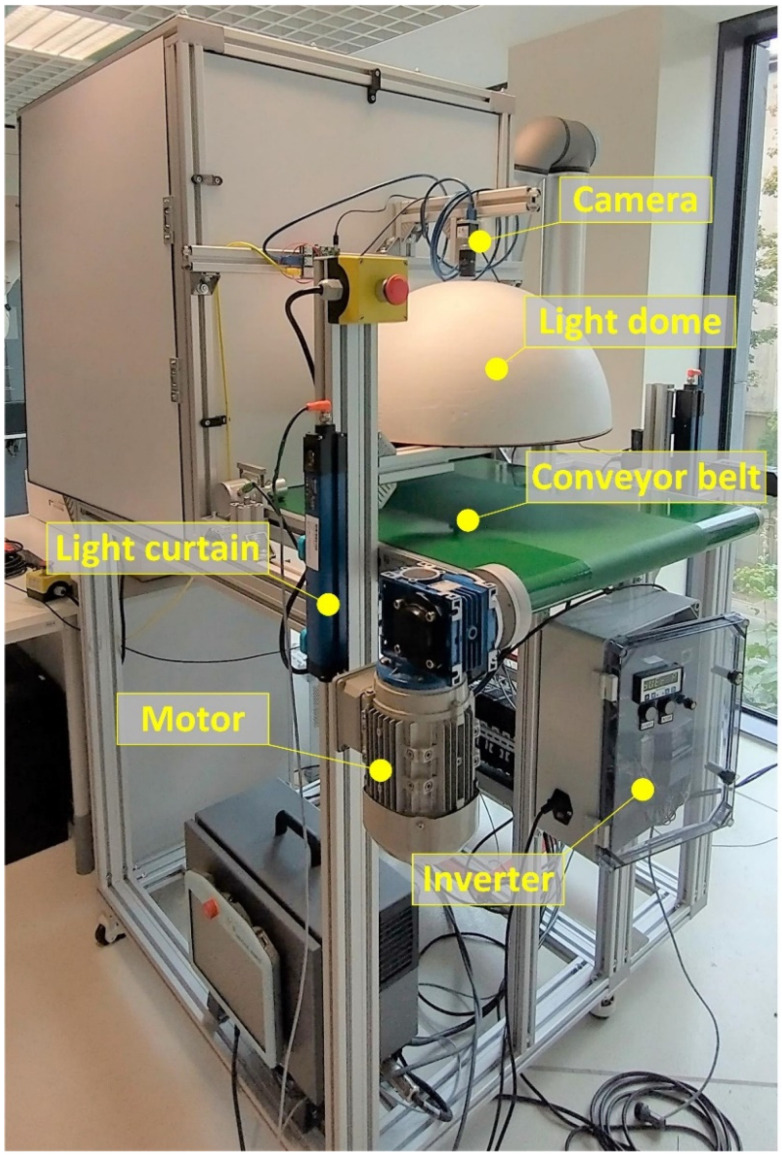
The laboratory stand.

**Figure 3:**
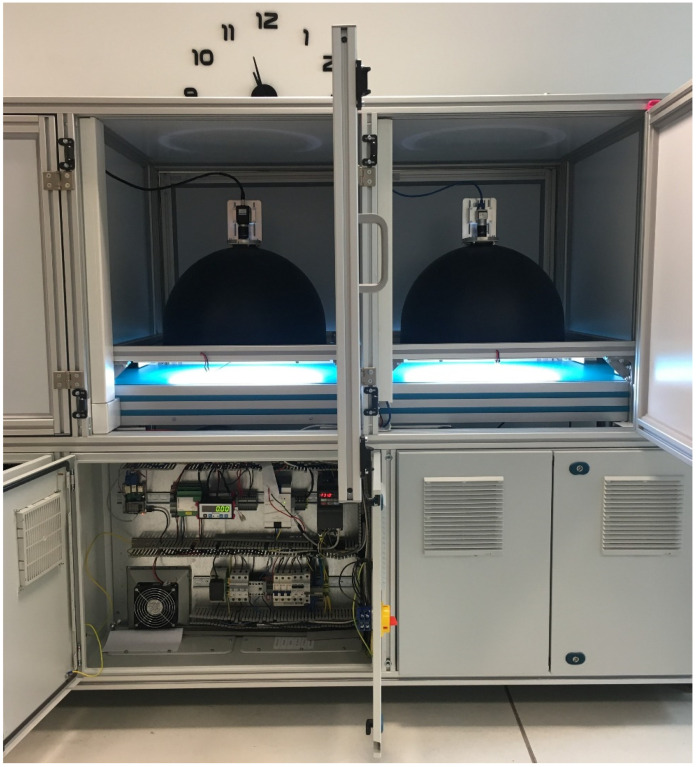
The industrial prototype of the inclusion detector. In order to speed up the process, the classifier and the detector are separate, so the object is already identified when it enters the detector module.

The laboratory stand used for the research is shown in Figure 2 and the prototype industrial device is presented in Figure 3. The hardware components of these test installations were adjusted to the requirements imposed on the project but can also be used as a starting point for the development of any optical control system with similar functionality but different specifications. In this section, a prototype is presented. Additionally, the issues related to its adjustment to the requirements and limitations of other applications are discussed.

The laboratory stand presented in Figure 2 consists of a conveyor belt driven by an electric motor controlled by an inverter. The belt speed is about 20 cm/s. Above the belt is a frame with the high-performance 5-megapixel monochrome CMOS camera (CA-H500MX, Keyence, Osaka, Japan) with a low distortion 12 mm lens (CA-LHR12) and a spherical dome light with an LED illumination ring. 

Based on experience gained during laboratory tests, a prototype industrial device was built, as presented in Figure 3. The main electromechanical components were copied directly from the laboratory stand, e.g., the conveyor belt, the drive, and the light curtain. The vision system is divided into two components: one responsible for part classification, and the other for detecting inclusions. The first module utilizes a cost-effective camera Basler ace acA2440-35uc with Sony CMOS 5 (Basler headquarters, Ahrensburg, Germany) megapixel color, a global shutter sensor, and the single-board computer Raspberry Pi 4 (Raspberry Pi Holdings, headquarters in Cambridge, UK) running a SqueezeNet-based algorithm as a tailored solution to classify parts, described in Section 2.4.1. The inclusion detection system is based on the same sensor as in the laboratory stand system, i.e., a high-performance 5-megapixel monochrome CMOS camera CA-H500MX with a low-distortion 12 mm lens CA-LHR12. 

**Figure 4:**
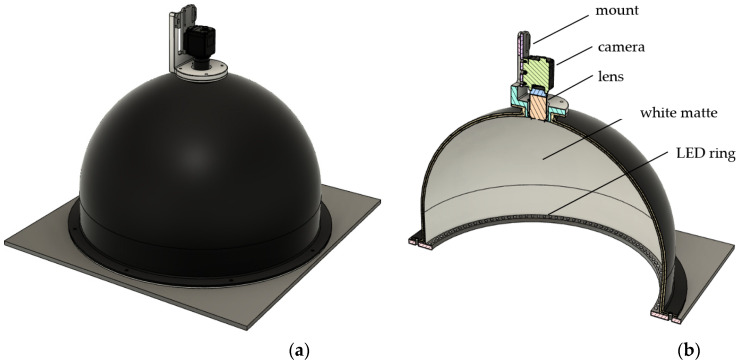
View of the dome light CAD model (**a**) and its cross-section with the interior visible, especially the light source (**b**).

A significant problem in the practical application of the inclusion detection method is shadows and reflections in the camera image. For this reason, appropriate diffuse illumination is essential in industrial optical inspection tasks involving shiny or complex surface details that require uniform visibility. Both modules of the prototype are equipped with dome lights, which produce non-directional reflected light (Figure 4) and effectively reduce glare and shadows. The domes were designed and produced to meet the requirements of the project. To achieve uniform illumination, each dome is a combination of a hemisphere and a cylinder. It is made of laminate, coated inside with a white matte finish, and equipped with an LED light source facing up that illuminates objects with reflected light. The exterior is coated with a black finish. The dome is equipped with a precision mount for a camera directly above the oculus.

The diameter of the dome light must be adjusted to the maximum size of the analyzed detail and the mounting height and focal length of the camera must ensure that the dome walls are behind the field of view. The industrial prototype uses a dome light with a diameter of 50 cm, which ensures uniform illumination of the polymer details with a maximum size of 24 × 24 cm. 

The first dome light prototype was made of a polystyrene hollow hemisphere covered inside with matte paper. The images captured with it were of sufficient quality for the presented defect detection algorithm to operate correctly, so such a dome light may be considered as a low-budget initial solution. 

The camera positioning, resolution, and focal length were chosen based on the maximum size of elements under inspection and desired accuracy. For the presented application, the height of the camera installation is 38 cm, which results in a field of view of 24 × 28 cm. The parameters of the system should be adjusted to the requirements of the specific application.

In the final solution, the classifier and the inclusion detector were separated (Figure 3). This approach speeds up detection and ensures real-time operation. The described algorithm has been implemented on the Keyence XG-X2900 vision controller. The average time of defect detection is 210 ms.

An important problem that should be considered is the triggering system, which initiates taking a picture at the right moment, based on the position of the element on the conveyor belt. A practical solution is to use an incremental encoder and a photosensor that detects the element at the beginning of the conveyor belt. For the assumed maximum conveyor speed of 20 cm/s, and the trigger precision of 500 μs, generated by a cyclic interrupt of the PLC, the maximum distance error is 100 μm.

### 2.2. Diagram of the Proposed Method

Inclusions can be detected as local irregularities on the surface of the object. The main problem is related to the edges of the object, which should be excluded from the calculations. Edge detectors cannot be directly applied to the object under inspection because some inclusions would be classified as edges and therefore excluded from the inclusion detection procedure. Thus, an edge mask prepared at the off-line stage was used and matched with the camera image. 

**Figure 5:**
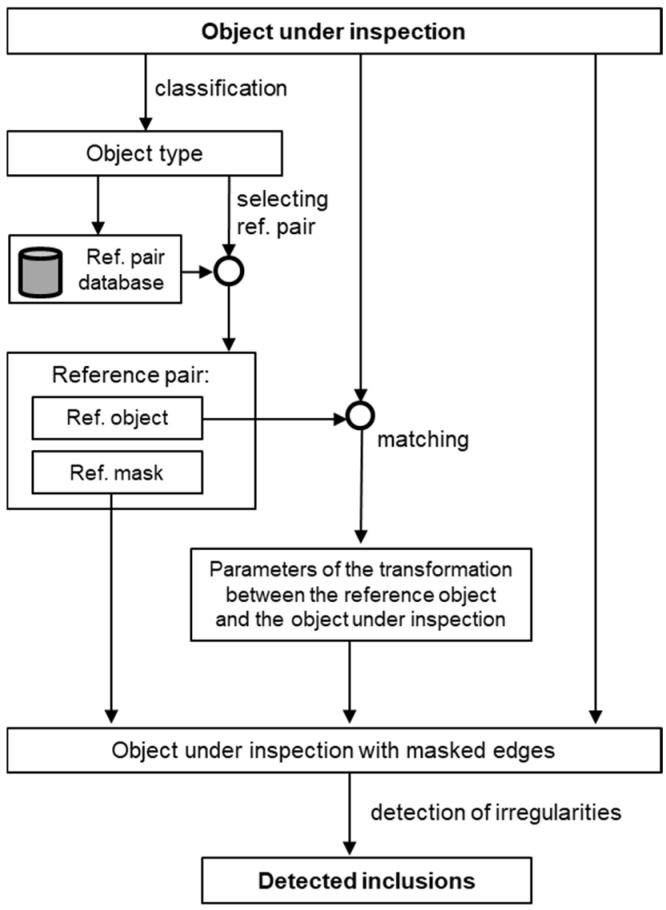
Diagram of the proposed method.

The diagram of the proposed method is presented in Figure 5. At the off-line stage, the reference pair database should be prepared. It contains two images for each element: the image of the element and the mask of edges. The algorithm for the preparation of the reference pair database is presented in Section 2.3. At the on-line stage, the object under inspection is classified—see Section 2.4 for the details—and matched with the corresponding reference image. The purpose of matching is to find parameters of transformation, composed of translation, rotation, and scaling, between the inspected object and the reference mask. Since the matching algorithm should receive two grayscale images as the input, the image of the object under inspection cannot be directly matched with the binary reference mask. Instead, it is matched with the reference image, which has the same parameters of translation, rotation, and scaling as the reference mask. After finding the parameters of transformation, the reference mask is transformed and superimposed with the image under inspection. For the part of the object surface that is not masked, local standard deviation is calculated. Areas where it exceeds the threshold value are considered inclusions.

### 2.3. Preparation of the Reference Pair Database

Inclusions are detected as areas of the object surface with a grayscale value different from the grayscale of the surrounding area. In order to avoid false alarms at object edges, in the first step, all edges are masked to exclude them from calculations. The mask is prepared off-line for each object class, together with the corresponding reference image, which has the same position, rotation, and scaling. An approach based on reference pairs consisting of the reference image and the reference mask aligned with the reference image was proposed. The reason is that the automatic adjustment algorithms require two grayscale images for correct operation. During the inspection process, the correct transformation is found by matching the reference image with the image under inspection. Parameters of this transformation are then used to superimpose the reference mask with the query image. 

For this reason, a reference pair should be prepared for each element. The pair contains the grayscale image of the element and the corresponding edge mask. Reference pairs can be prepared off-line, once for every new element added to the database. For localization of edges, two alternative methods were designed and implemented:

1. Edge mask calculation directly from the reference image
  In the first step, edges are detected using the Canny filter. Parameters of the filter, particularly standard deviation and hysteresis thresholds, are adjusted to ensure correct detection and avoid false detections, which would cause the exclusion of some areas from the inclusion detection algorithm. Then, morphological filters [23] are applied in order to extend the mask to the neighborhood of edges detected by the Canny filter.
2. Edge mask calculation from the 3D model
  The method requires a 3D model of the object. The corresponding projection of the model edges is superimposed on the object image from the camera. This method is more robust than the first one, as it is not sensitive to noise, shadows, etc. However, it requires matching the model with the object observed by the camera.

In both methods, it is assumed that the result is verified. In some cases, automatically calculated edge masks are flawless, but manual correction is often necessary, which can be performed in a graphical editor such as InkScape or Gimp. The reasons for imperfect operation are explained at the end of this section. 

#### 2.3.1. Method 1: Edge Mask Calculation Directly from the Reference Image

The algorithm includes the following steps:

1. Edge detection using a Canny filter. A relatively high standard deviation allows for discarding small irregularities, which should not be included in the edge mask as potential flaws.
2. Dilation of edges using a circle with diameter *d_d_* as a structuring element. The purpose is to exclude from calculations not only one-pixel-wide edges but also the surrounding area, since in later stages irregularities are searched for in the neighborhood of each non-masked pixel of the object.
3. Inclusion in the edge mask of small, isolated areas surrounded by edges, which are assumed to be narrow hollow areas or small poles, where shadows can often be present and may cause false alarms. This step is performed as morphological closing using a circle with diameter *d_c_* as a structuring element.
  Exclusion from the edge mask of small, isolated areas, which may be potential inclusions that appeared at the output of the Canny filter. This is achieved by removing all connected components that have fewer than *p* pixels.

The mask can be described as a binary image *M*:

*M* = *E* ⊕ *C_dd_* • *C_dc_*, (1)

where *E* is the image of edges, *C_dd_* is a circle with radius *d_d_*, and *D_dc_* is a circle with radius *d_d_*. Operators ⊕ and • denote morphological operations of dilation and closing, respectively. 

Based on experiments, the following values of algorithm parameters were used:

- Standard deviation of Canny filter: *σ* = 5;
- Hysteresis thresholds of Canny filter: *Θ*_1_ = 0.02, *Θ*_2_ = 0.1;
- Size of the structuring element for dilation in Step 2: *d_d_* = 15;
- Size of the structuring element for closing in Step 3: *d_c_* = 10;
- Minimum number of pixels for connected components: *p* = 300.

**Figure 6:**
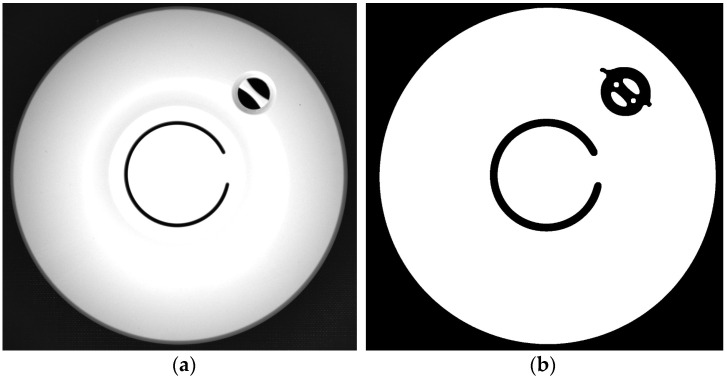
Original image (**a**) and the edge mask calculated fully automatically based on the edge detector (**b**). The mask defines the area where inclusion detection will be performed.

**Figure 7:**
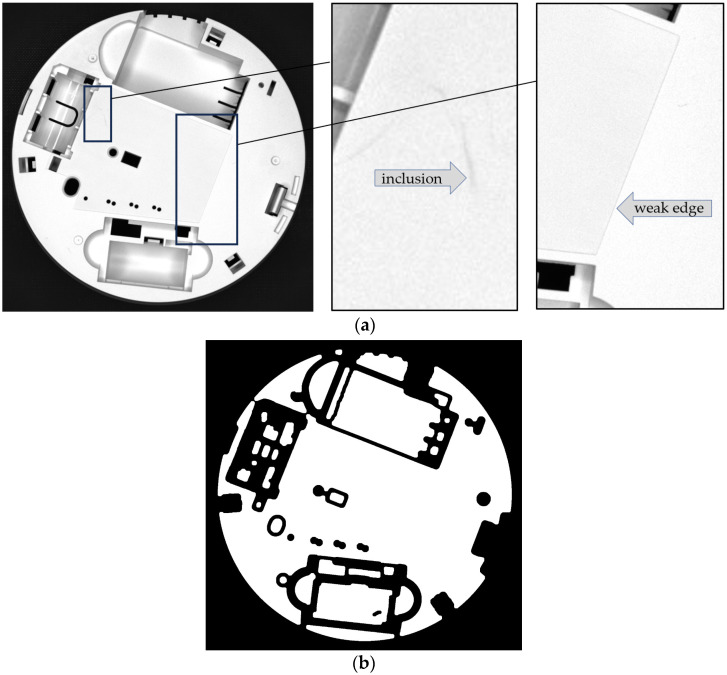
Example of situation where some edges—in this case, the edge of the quadrangle indicated with the arrow—were not properly detected. Figure (**a**) presents the camera image and figure (**b**) the corresponding edge mask.

Two examples of edge masks calculated using this algorithm are presented in Figure 6 and Figure 7. The quality of the results is satisfactory, but in both masks, there are some issues that should be corrected manually. The edge forming a quadrangle indicated with the arrow in Figure 7a was not correctly detected. Increasing the sensitivity of the edge detector would allow this edge to be masked, but would bring the risk of masking some flaws, which therefore could not be detected by the inclusion detection algorithm. 

#### 2.3.2. Method 2: Edge Mask Calculated from the 3D Model

One of the functionalities of the quality control system is the control of product geometry. This is beyond the scope of the paper, but 3D post-production models that were created using the GOM Atos Scanbox in order to control the geometry of parts can also be used for the alternative method for creating edge masks, as described in this section. 

The first step of the algorithm is to prepare the 2D projection of edges from the three-dimensional model saved in the STL file. The next step is to prepare the reference pair:

- The reference image, which is an image of the object from the camera, taken, if possible, under similar conditions (illumination, camera parameters) to the target conditions;
- The reference mask—the binary mask of edges calculated from the STL file, aligned with the reference image based on a manual indication of corresponding points.

**Figure 8:**
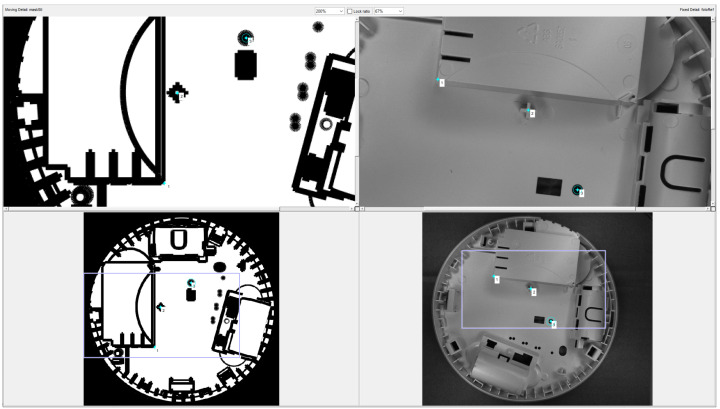
A screenshot from the application for matching the mask calculated from the STL file (**bottom left**) with the reference image (**bottom right**). Top images are magnified fragments of the bottom images, used for navigation. The corresponding points are labelled with their numbers.

Figure 8 presents a screenshot from the application for matching the mask calculated from the 3D model with the reference image. The user marks the corresponding points on both images with the mouse and based on these points, the parameters of the geometric transformation are calculated. 

**Figure 9:**
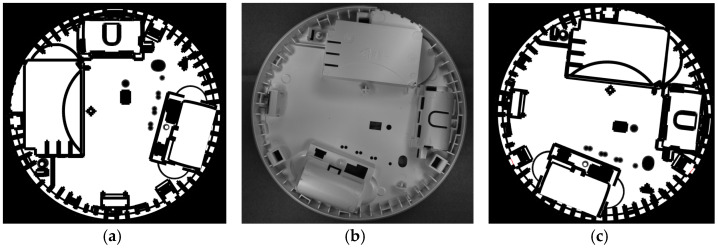
The mask calculated from the STL file (**a**), the reference image (**b**), and the reference mask (**c**).

Figure 9 presents the matching result: the mask calculated from the 3D model (left), and the reference pair: the reference image (middle) and the reference mask (right), i.e., the mask after alignment with the reference image.

#### 2.3.3. Comparison of Edge Mask Calculation Methods

The two methods for edge mask calculation presented in this section were compared in terms of three criteria: accuracy, automation, and robustness. The first method, based on the edge detector, is very fast and fully automated, but it gives lower accuracy. In order to ensure sufficient robustness, the detected edges must be heavily dilated. Also, any noise coming from the edge detection filter results in unnecessary exclusion of some areas from quality control. The second method, based on 3D models, is more accurate but it requires an additional manual action—indicating at least three corresponding points to calculate the geometric transformation. 

Some types of parts have very complex shapes, which is sometimes challenging for automatic mask calculation and requires manual adjustments. Other types of parts have elements that are not present in the CAD 3D models (e.g., injection marks) and have to be masked manually. Regardless of the method used, the manual inspection and correction of the calculated edge mask is assumed. This action should be carried out only once for each part at the stage of database creation. The comparison of the two proposed methods is summarized in Table 1.

**Figure 10:**
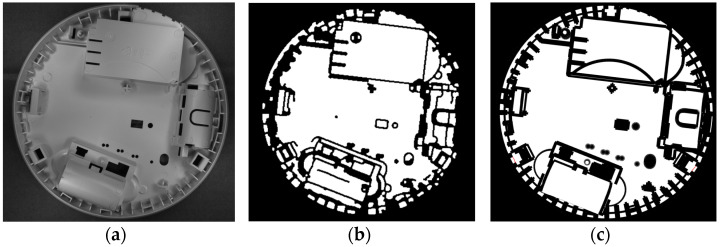
An element particularly difficult for edge detection (**a**), its edge mask for the same element calculated from the reference image (**b**), and from the STL file (**c**).

Figure 10 presents the comparison of the edge masks calculated for the same element (a) using direct calculation from the reference image (b) and from the STL model (c). 

The element in Figure 10 was particularly difficult for edge detection due to its complex shape and many shadows. It can be noticed that the quality of the STL-based method is considerably higher. 

### 2.4. Inclusion Detection

The section presents the on-line part of the process, related to inclusion detection during the production process. Inclusion detection includes three stages:

- Part classification, necessary to apply the corresponding edge mask; see Section 2.4.1;
- Matching the reference mask with the image under inspection; see Section 2.4.2;
- Detection of irregularities in the surface grayscale, performed for the part of the object surface that has not been masked by the edge mask; see Section 2.4.3.

#### 2.4.1. Part Classification

The set of elements for inclusion detection contains different types of parts. The elements differ in shape and size, but the color is similar—creamy white. Information about which type of part is coming to the detection subsystem is crucial for matching the reference image. Therefore, the stand was equipped with a classifier that placed each part entering the detection subsystem into one of the known classes based on an image from the camera.

**Figure 11:**
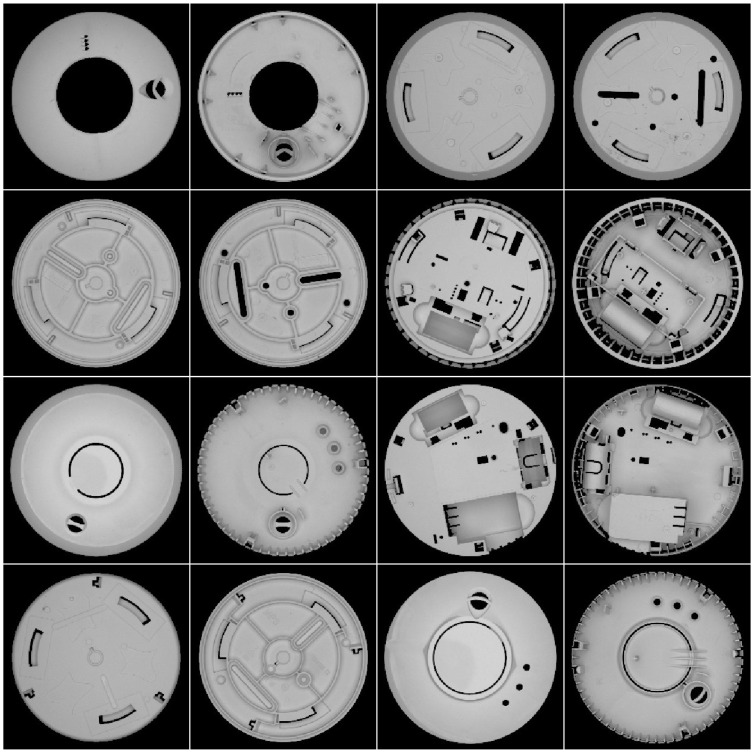
Samples of the training set. Each part is shown on both sides.

The classifier algorithm is based on SqueezeNet—a relatively fast and light convolutional neural network [24]. Relatively good performance of this network, comparable to AlexNet and sufficient for the application, was achieved with merely 5 MB of parameters. Transfer learning, where a pre-trained SqueezeNet model was used as a starting point for the training process, was used. The network was trained on a set of images registered from 15 types of double-sided parts, which gives 30 classes. The training set, which included 10 images per class, was augmented by random rotation and translation, which gave a total of 1000 pictures in each class. A few samples of that training set are depicted in Figure 11. 

The training of 20 epochs took less than a minute on a PC. For the database of 30 classes, faultless classification was achieved.

#### 2.4.2. Matching the Reference Mask with the Image Under Inspection

The use of the reference mask requires its geometric matching with the object. For this purpose, in the off-line stage, the database with reference pairs was prepared. Each reference pair contains:

- The reference object, i.e., the object seen by the camera in a fixed position, called the reference position;
- The reference mask, i.e., a mask calculated from a 3D object geometrically transformed to a position identical to the position of the reference object.

Matching the reference mask with the image under inspection consists of two stages:

Stage 1: Finding the transformation parameters by matching the reference image with the camera image. 

The purpose of this stage is to find parameters Ψ* of the reference image transformation that maximize the similarity measure *s* between the camera image *I* and transformed reference image *T_Ψ_* (*R*):

Ψ* = argmax *s*(*I*, *T_Ψ_* (*R*)), (2)

where *Ψ* is a vector of transformation parameters:

*Ψ* = (Δ*x*, Δ*y*, *ρ*, *α*). (3)

and Δ*x*, Δ*y*, *ρ*, *α* denote transformation parameters: translation, scale, and rotation. *T_Ψ_* denotes transformation with parameter vector *Ψ*. The adjustment is made using the SURF method [25] and the similarity measure *s* is based on the similarity of the corresponding keypoints.

Stage 2: Application of the transformation with the parameters found in stage 1 to superimpose a reference mask on the object observed in the camera. 

The masked image *I_M_* is calculated as an intersection of the camera image *I* and the complement of the transformed mask image:

(4) $$I_{M}=I\;\cap \;\bar{T_{Y}\left(M\right)}.$$

**Figure 12:**
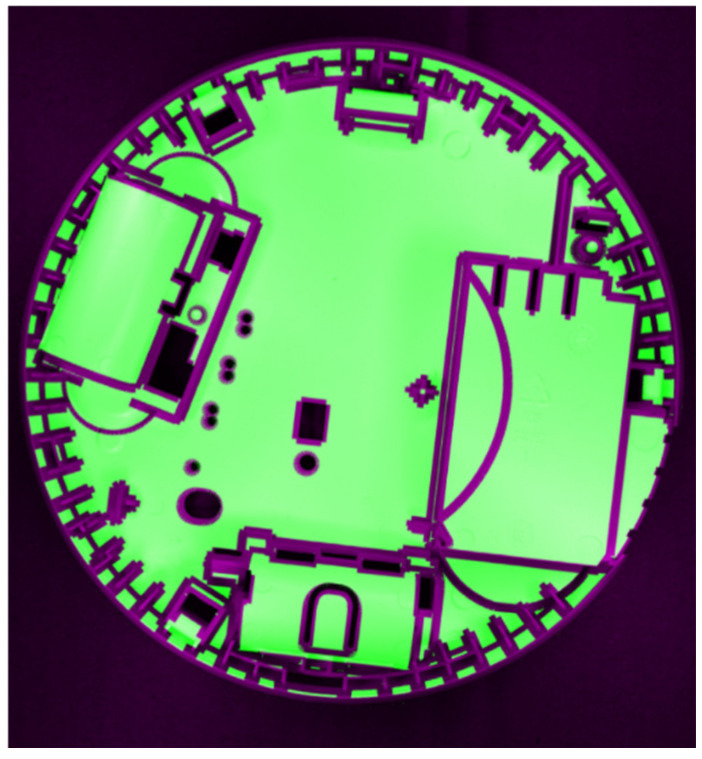
The reference mask superimposed on the image observed by the camera.

The final effect of stage 2 is presented in Figure 12.

After matching the reference mask with the image under inspection, the irregularities in the surface grayscale can be detected in the masked image.

#### 2.4.3. Detection of Irregularities in the Surface Grayscale

After matching the reference mask with the image under inspection, the surface irregularities detection algorithm is triggered. Areas with a high local standard deviation are indicated as inclusions. First, the image is subjected to median filtering with a *d_m_* × *d_m_* window, and then the value of the local standard deviation in the moving *d_s_* × *d_s_* window is calculated. Good results were obtained for 3 × 3 median filtering and a standard deviation window size of 11 × 11 pixels.

**Figure 13:**
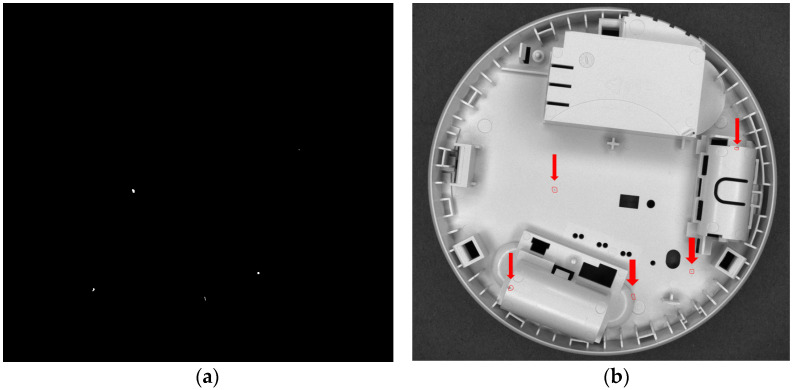
Final result—areas where, in all photos with side lighting, the local standard deviation exceeds the threshold value (**a**), and these areas (indicated by red circles and arrows) superimposed on the original photo (**b**).

The output image from the local standard deviation filter is then thresholded with threshold *Θ_s_* and areas along the edge are excluded. Inclusions are detected for pixels fulfilling the following condition:

*F_s_*(*F_m_*(*I*)) > *Θ_s_* ∩ ~*M*, (5)

where *I* is the camera image, *F_m_* denotes median filtering, *F_s_* is the local standard deviation filter, *Θ_s_* is the threshold, and *M* is the mask. The result is presented in Figure 13, where the detections are superimposed on the original photo.

**Figure 14:**
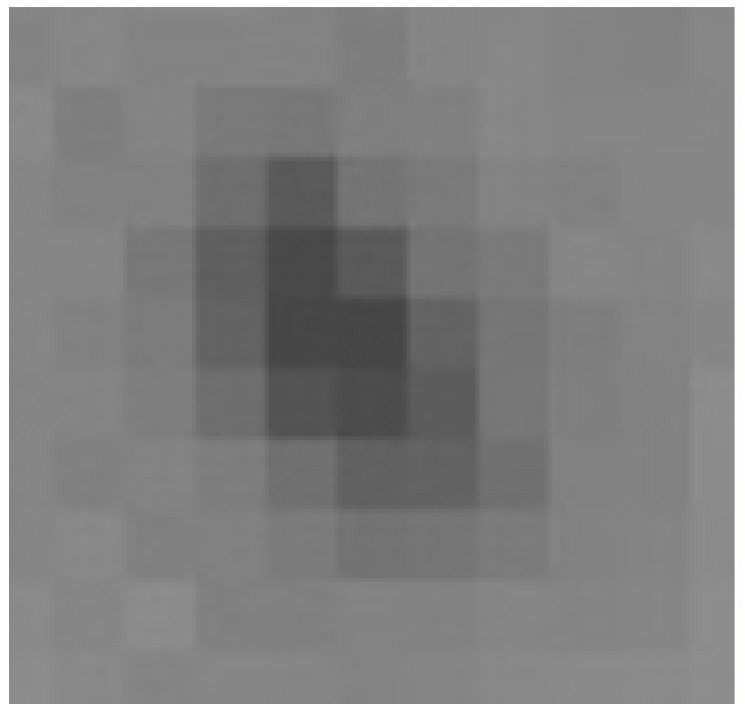
The smallest detected inclusion—one pixel in this photo is 0.0702 mm.

Figure 14 shows the smallest detected inclusion. The diameter of the inclusion presented in Figure 14 is around 5 pixels, which corresponds to 0.35 mm.

## 3. Results and Discussions

**Figure 15:**
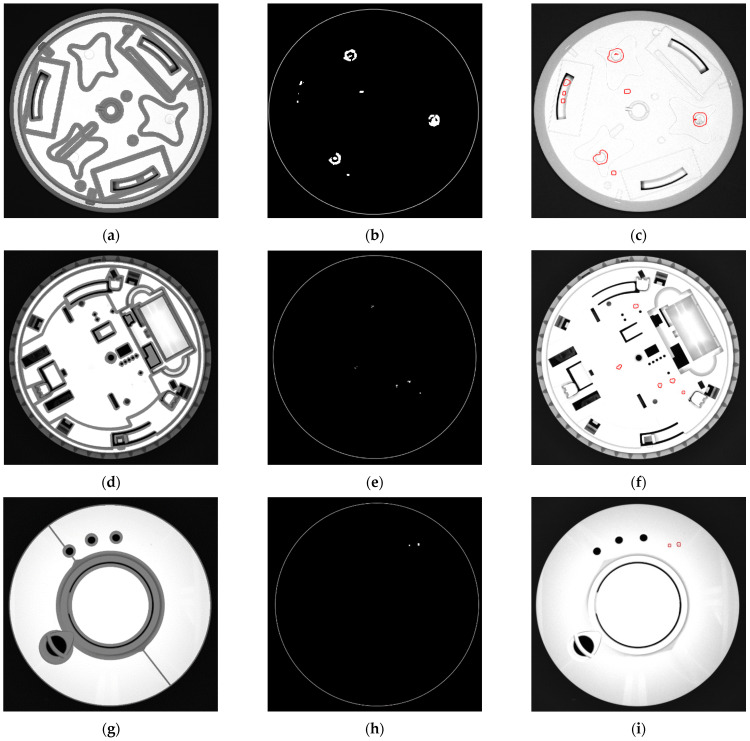
Sample results of inclusion detection for three different parts. Each row corresponds to the same part. Left column (**a**,**d**,**g**) shows images of parts with their masks superimposed. The middle column (**b**,**e**,**h**) shows detected inclusions. Right column (**c**,**f**,**i**) shows the parts with inclusions drawn in red.

The first test of the algorithm was conducted on 15 types of double-sided parts moving on the conveyor, with edge masks calculated from 3D models. However, it was noticed that effectiveness strongly depends on proper mask preparation. Since the edges were derived from CAD models, initially the edge masks did not contain artifacts such as injection marks resulting from the technological process of molding. There is an example of such artifacts in the first row of Figure 15—three big irregularities identified as inclusions come from injection marks. However, these irregularities should be treated as normal, so the masks need to be manually corrected to extend the masked area to the injection marks.

For the quantitative assessment of the algorithm, a database with 79 images of parts with identified inclusions was prepared in cooperation with CBRTP experts. The total number of inclusions was 261. 

Edge masks for this experiment were calculated automatically from the 2D image, using method 1 described in Section 2.3.1 with parameters: σ = 5, *Θ*_1_ = 0.02, *Θ*_2_ = 0.1, *d_d_* = 15, *d_c_* = 10, and *p* = 300. The parameters of the detection algorithm were tuned to ensure that all inclusions would be detected, even if high sensitivity would cause some false alarms. This was achieved for the following parameter values: *F_m_* = 3, *F_s_* = 11, and *Θ_s_* = 5. In the experiment, all inclusions were detected. In addition, there were 40 detections in spots where the experts did not notice any inclusions. These 40 additional detections are caused by existing variations in grayscale but variations are imperceptible and would not normally be the reason for rejecting the product, and therefore can be classified as false detections. For the assessment, common measures in image detection systems: recall, which is in the presented case the percentage of inclusions that were detected, and precision, which says what percentage of detections are inclusions and not false alarms were utilized. Standard indicators used for the assessment of automatic detection systems, their description in the context of this application, and their values are presented in Table 2.

By adequate tuning of parameters, 100% recall on the testing set used in experiments was obtained. Such a result is very important in the quality control system. Even several flawed parts may result in the rejection of the whole consignment by the client and bring large losses to the company. High precision is in such cases much less important. The obtained 87% precision means that 13% of inclusion detections are false alarms. In the case of the proposed target application, this is acceptable, especially because the rejected parts can be later inspected manually. 

Due to the requirements of the target application, the algorithm is tuned to eliminate undetected inclusions (false negatives) at the cost of some false alarms. In conducted experiments, 100% recall with precision at the level of 87% was achieved. This result is acceptable and makes the algorithm useful for industry. 

Summarizing, the paper describes a novel method for the detection of flaws in plastic objects coming from injection-molding machines, which are visible on the surface of products as blobs with a different color, usually darker than the rest of the surface. The prototype of the commercial version was tested in an industrial environment and proved its effectiveness. Future research will be focused on commercialization and adapting to other types of products. 

## Figures and Tables

**Table 1:** Performance comparison of the methods for edge mask calculations.

	Method Based on Edge Detector	Method Based on 3D Model
Accuracy	Low Sensitive to the noise from the edge detection filter. The flaws recognized by the filter as edges cannot be detected and undetected edges can be later reported as flaws.	Medium Parts details, such as injection marks, not included in CAD models may cause false detections.
Automation	High Fast and fully automated, no manual action needed.	Medium Needs some manual adjustment for matching the mask with the reference image, when a model is introduced to the database.
Robustness	Low Sensitive to edge detection filter parameters, the results depend on lighting conditions, noise, and quality of the image. Needs heavily dilated edges—some areas have to be excluded from quality control.	High No need to detect edges, which are always well matched and excluded from quality control.

**Table 2:** Performance comparison of the methods for edge mask calculations.

Indicator	Description	Value
TP (true positive)	The number of inclusions that were correctly detected	261
FP (false positive)	The number of false detections—when grayscale variation is detected as inclusion, but human experts consider the sample acceptable	40
FN (false negative)	The number of undetected inclusions	0
Precision	TP/(TP + FP) Percentage of detections that are classified by experts as inclusions	261/(261 + 40) = 87%
Recall	TP/(TP + FN) Percentage of inclusions that were detected	261/(261 + 0) = 100%

## Data Availability

Restrictions apply to the availability of these data. Data were obtained from Hanplast Sp. z o.o. and CBRTP S.A. and are available with the permission of Hanplast Sp. z o.o. and CBRTP S.A.

## References

[1] Luo Q., Fang X., Su J., Zhou J., Zhou B., Yang C., Liu L., Gui W., Tian L. (2020). Automated Visual Defect Classification for Flat Steel Surface: A Survey. IEEE Trans. Instrum. Meas..

[2] Fang X., Luo Q., Zhou B., Li C., Tian L., Fang X., Luo Q., Zhou B., Li C., Tian L. (2020). Research Progress of Automated Visual Surface Defect Detection for Industrial Metal Planar Materials. Sensors.

[3] Xie X. (2008). A Review of Recent Advances in Surface Defect Detection using Texture analysis Techniques. ELCVIA Electron. Lett. Comput. Vis. Image Anal..

[4] Tsai D.M., Chen M.C., Li W.C., Chiu W.Y. (2012). A fast regularity measure for surface defect detection. Mach. Vis. Appl..

[5] Ma Y., Li Q., Zhou Y., He F., Xi S. (2017). A surface defects inspection method based on multidirectional gray-level fluctuation. Int. J. Adv. Robot. Syst..

[6] Weyrich M., Wang Y. (2011). A Real-time and Vision-based Methodology for Processing 3D Objects on a Conveyor Belt Model Driven Development of Service Oriented Plant Controls View project Autonomous Systems View project. Int. J. Syst. Appl. Eng. Dev..

[7] Zhiznyakov A.L., Privezentsev D.G., Zakharov A.A. (2015). Using fractal features of digital images for the detection of surface defects. Pattern Recognit. Image Anal..

[8] Zhang M., Shi H., Yu Y., Zhou M. (2020). A computer vision based conveyor deviation detection system. Appl. Sci..

[9] Yang Y., Miao C., Li X., Mei X. (2014). On-line conveyor belts inspection based on machine vision. Optik.

[10] Ren Z., Fang F., Yan N., Wu Y. (2021). State of the Art in Defect Detection Based on Machine Vision. Int. J. Precis. Eng. Manuf.-Green Technol..

[11] Bhatt P.M., Malhan R.K., Rajendran P., Shah B.C., Thakar S., Yoon Y.J., Gupta S.K. (2021). Image-Based Surface Defect Detection Using Deep Learning: A Review. J. Comput. Inf. Sci. Eng..

[12] Ke K.C., Huang M.S. (2020). Quality prediction for injection molding by using a multilayer perceptron neural network. Polymers.

[13] Cha Y.-J., Choi W., Suh G., Mahmoudkhani S., Büyüköztürk O. (2018). Autonomous Structural Visual Inspection Using Region-Based Deep Learning for Detecting Multiple Damage Types. Comput.-Aided Civ. Infrastruct. Eng..

[14] Kocon M., Malesa M., Rapcewicz J. (2024). Ultra-Lightweight Fast Anomaly Detectors for Industrial Applications. Sensors.

[15] Zong Y., Liang J., Wang H., Ren M., Zhang M., Li W., Lu W., Ye M. (2021). An intelligent and automated 3D surface defect detection system for quantitative 3D estimation and feature classification of material surface defects. Opt. Lasers Eng..

[16] Chen Y., Ding Y., Zhao F., Zhang E., Wu Z., Shao L. (2021). Surface defect detection methods for industrial products: A review. Appl. Sci..

[17] Liu L., Wang H., Yu B., Xu Y., Shen J. (2007). Improved algorithm of light scattering by a coated sphere. China Particuology.

[18] Li B., Wang J., Gao Z., Gao N. (2021). Light Source Layout Optimization Strategy Based on Improved Artificial Bee Colony Algorithm. Math. Probl. Eng..

[19] Kokka A., Pulli T., Ferrero A., Dekker P., Thorseth A., Kliment P., Klej A., Gerloff T., Ludwig K., Poikonen T. (2019). Validation of the fisheye camera method for spatial non-uniformity corrections in luminous flux measurements with integrating spheres. Metrologia.

[20] Kokka A., Pulli T., Poikonen T., Askola J., Ikonen E. (2017). Fisheye camera method for spatial non-uniformity corrections in luminous flux measurements with integrating spheres. Metrologia.

[21] Canny J. (1986). A Computational Approach to Edge Detection. IEEE Trans. Pattern Anal. Mach. Intell..

[22] Granlund G.H. (1978). In Search of a General Picture Processing Operator. Comput. Graph. Image Process..

[23] Serra J. (1982). Image Analysis and Mathematical Morphology.

[24] Iandola F.N., Moskewicz M.W., Ashraf K., Han S., Dally W.J., Keutzer K. SqueezeNet: AlexNet-level accuracy with 50x fewer parameters and <1 MB model size. Proceedings of the ICLR.

[25] Bay H., Ess A., Tuytelaars T., Van Gool L. (2008). SURF: Speeded Up Robust Features. Comput. Vis. Image Underst. (CVIU).

